# Style: J of occupational medicine and toxicology vibration induced injuries in hands in long-term vibration exposed workers

**DOI:** 10.1186/s12995-019-0242-0

**Published:** 2019-07-15

**Authors:** Lars Gerhardsson, Mats Hagberg

**Affiliations:** Occupational and Environmental Medicine, University of Gothenburg, Medicinaregatan 16, Box 414, SE-405 30 Gothenburg, Sweden

**Keywords:** Hand-arm vibration, Neuropathy, Quantitative sensory testing, Dominant and, Non-dominant hand

## Abstract

**Introduction:**

Long-term vibration exposure may cause neurophysiological disturbances such as numbness and tingling, reduced grip strength and difficulties in handling small objects. The dominant hand will usually have a higher vibration exposure than the non-dominant hand, which may cause more severe neurological symptoms and signs in the dominant hand.

**Methods:**

The study is based on 47 (36 males and 11 females) vibration exposed workers, all former patients from the department of Occupational and Environmental medicine, Gothenburg university. The comparison group consisted of 18 randomly selected subjects from the general population of Gothenburg. All participants completed several questionnaires and had a standardized medical examination. Thereafter, neurophysiological tests such as the determination of vibration and thermal perception thresholds were performed, as well as muscle strength tests in hands and fingers.

**Results:**

The temperature perception thresholds (TPTs) and the vibration perception thresholds (VPTs) did not differ significantly between the dominant and non-dominant hand in vibration exposed workers. The referents showed a significantly better performance (*p* ≤ 0.02 and *p* ≤ 0.034, respectively) than the workers for both TPTs and VPTs, indicating a negative effect on the Aß, as well as on the Aδ and C-fibers among the exposed workers.

The Purdue Pegboard test showed a significantly better performance in the dominant vs non-dominant hand in both workers (*p* = 0.001) and referents (*p* = 0.033). The referents showed a better performance than the workers in both hands (*p* < 0.001). The Baseline handgrip, the Pinch grip and 3-Chuck grip tests did not differ significantly between the dominant and non-dominant hand in neither workers nor referents.

**Conclusions:**

In this study, minor differences between the dominant and non-dominant hand were noted for the Purdue Pegboard test in both workers and referents. Despite a probably higher vibration exposure in the dominant hand (mostly the right hand), however, quite similar test results were noted for VPTs, TPTs, Baseline handgrip, Pinch grip and 3-Chuck grip when comparing the dominant and non-dominant hand in the vibration exposed workers. In case of lack of time and financial obstacles, neurological tests in solely the dominant hand, will probably satisfactory reflect the conditions in the non-dominant hand.

## Background

Hand-arm vibration exposure is still common in working life. In Sweden it is estimated that about 400 000 workers have a daily exposure to vibrating tools exceeding 2 h. Common symptoms after long-term vibration exposure include Raynaud’s syndrome (vibration white fingers, VWF) and neurophysiological disturbances such as numbness and tingling, reduced grip strength and difficulties in handling small objects like coins etc. [1]. Several neurophysiological tests can be used for the diagnosis of vibration-induced neuropathy. Two diagnostic corner stones are the determination of thermotactile and vibrotactile perception thresholds [2]. The hot and cold thresholds reflect the function of warm and cold receptors, while vibrotactile thresholds at 31.5 and 125 Hz assess the function of the Meissner and Pacinian corpuscles. These tests are usually performed on the distal phalanges of the index finger and little finger on both hands to mirror the function of the median and ulnar nerves.

There are many different types of vibrating tools with different weights and acceleration amplitudes. Several studies have shown that it is difficult for a worker to accurately estimate the exposure time to vibrating tools. Usually there is an overestimate, which may vary from two and even up to eight times [3]. This uncertainty complicates the calculation of the correct vibration dose. Another complicating factor when performing a dose-response evaluation is the large variation in sensitivity to vibration among workers. Some workers will develop quite severe symptoms within just a few years, while other workers may work for decades without any major problems.

Smaller and lighter vibrating tools are usually held in the dominant hand, which thereby will have a higher vibration exposure than the non-dominant hand. For heavier tools both hands are usually used and for e.g. compact wrenches the non-dominant hand holding the anvil may sometimes get an even higher vibration exposure than the dominant hand.

In previous studies, the impact of psychological status on work ability in vibration exposed workers have been investigated [4]. The influence of other factors on work ability, e.g. age, gender, muscle pain and stress levels was also studied [5] as well as the test-retest reliability of neurophysiological tests that are used for the diagnosis of the hand-arm syndrome [6].

In this study we are comparing test results in the dominant and non-dominant hand in long-term vibration exposed workers for the determination of vibration perception thresholds, temperature perception thresholds, muscle strength tests in hands and fingers and eye-hand coordination (Purdue Pegboard test). The underlying hypothesis is that signs of adverse health effects will be more pronounced in the dominant hand, which in most cases will have the highest vibration exposure.

## Materials and methods

The study is based on 47 (36 males and 11 females; mean-age 50 ± 12 y; mean exposure time 16 y) vibration exposed workers, all former patients from the department of occupational and environmental medicine, Gothenburg university, where they since 2005 have been investigated because of vibration related symptoms and signs.

The exposed group had a mixed exposure to different types of vibrating, handheld tools, e.g. concrete breakers, sanders, grinders, disc cutters, drills, impact wrenches of different weights and sizes and screwdrivers. Several occupations were represented, e.g. construction industry, building and maintenance of roads, heavy engineering and motor vehicle manufacturing and repair. Lighter tools weighing up to around one kilogram have mainly been held in the dominant hand and heavier tools weighing 2–3 kg or more in both hands. The use of compact wrenches in the exposed group was relatively limited with short trigger times, which should only have had a marginal impact on the outcome of the study.

The comparison group consisted of 29 randomly selected subjects from the general population of Gothenburg, of which 18 (mean-age 37.6 ± 15.9 y) accepted to participate in the study.

After signing a written consent, the participants spent 3–4 h at our clinic to complete several questionnaires with questions about e.g. work and medical history, use of tobacco and alcohol, use of vibrating tools (years), symptoms related to vibration exposure (vibration white fingers, VWF; numbness and tingling) as well as questions about the general health status.

Thereafter, an experienced physician performed a standardized medical examination. The neurophysiological tests included Baseline handgrip strength, Pinch-grip and 3-Chuck grip (strength in finger muscles), determination of thermal (TPT) and vibration (VPT) perception thresholds and the Purdue Pegboard test.

The participants were asked to avoid vibration exposure during the day of the measurement. Coffee and tobacco had to be avoided at least 1 h before the medical tests. The ethical committee at the University of Gothenburg has approved the study.

The handgrip strength was performed by a Baseline® Hydraulic Hand Dynamometer (Fabrication Enterprises Incorporated, New York, NY, USA) through a standardized procedure using handle position number 2**.** The mean of three measurements was calculated for the dominant and non-dominant hand, respectively. For the determination of finger muscle strength a mechanical pinch gauge (PG-60; North Coast Medical, San José, CA, USA), was used [7]. The key-grip strength (Pinch key) and the three-digit pinch (Pinch 3-Chuck) were determined using the mean of three measurements in each hand.

### Vibrotactile measurements

Measurements of vibrotactile thresholds were evaluated by delivering sinusoidal vibrations to the pulp of digits 2 and 5 in both hands (the ascending-descending method of limits) and registering the subject’s response, using the VibroSense Meter® system (Vibrosense Dynamics, Malmö, Sweden). Sinusoidal frequencies at seven frequencies (8 Hz, 16 Hz, 32 Hz, 64 Hz, 128 Hz, 256 Hz, and 512 Hz), were delivered and transmitted to the finger pulp by a vibration probe (diameter 4 mm). The test did not start until the skin temperature of the subject’s forefinger exceeded + 28 °C**.** The contact force between the probe and the finger was 1 N and the forearm and the wrist of the participant was supported. The magnitude of the vibration was increased until the patient pressed the response button. The vibration magnitude was then decreased until the patient released the response button. Thereafter, the amplitude of the stimulus automatically began to rise again. The rate of change of the vibration amplitude was 3 dB/s and for each frequency there were six reversals. Thereafter, the testing automatically continued to the next frequency. The individual results were age-corrected [8] after comparison with values from a reference population supplied by the manufacturer of the equipment. All participants used ear protective devices to eliminate the noise from outdoor and indoor sources.

A sensibility index was calculated by dividing the integrated area under the obtained vibrogram curve of each object tested by that of the corresponding area under a superimposed and age matched reference curve. A sensibility index of less than 0.8 was regarded as the cut off value and indicates an abnormal response [9].

Measurements of vibration perception thresholds have shown a good to excellent reliability in studies of university and newspaper employees [10]. Also in subjects with diabetic neuropathy the determination of vibration perception thresholds have shown an excellent reliability, 0.85 [11] and ICC > 0.94 [12].

### Thermal thresholds

Quantitative testing of thermal sensibility was performed with an unidirectional stimulation technique using a commercially available test instrument with a Peltier element-based thermode of 25 × 50 mm (Termotest®; Somedic Sales AB). The tests were performed on the pulps of digits 2 and 5 on the dominant (DH) and non-dominant hand (NDH). The starting temperature was 32 °C for both cold and warmth, and the forearm and the wrist of the participant were supported. The perception thresholds to non-painful cold and warmth, respectively, were obtained by delivering six cold stimuli, followed by six warm stimuli in random order, at a rate of 1 °C/sec. The subject was instructed to press a button of a handheld switch at the first sensation of cold and warmth. The temperature then decreased or increased by 1 °C per second until the subject released the response button. The procedure was repeated another five times. The average of the last four assessments for cold and warmth on the finger pulps of digits 2 and 5 was calculated as the cold or warmth perception thresholds.

### Purdue pegboard test

The Purdue Pegboard test board (model 32020) from the Lafayette Instrument Company has two parallel rows with 25 holes into which cylindrical metal pegs should be placed, one by one, by the participants. After a brief practice, the dominant and non-dominant hand are tested three times each during a test period of 30 s. Thereafter, the mean score is calculated and compared with values from a reference population supplied by the manufacturer. The reliability is high. A three–trial administration test showed a high test-retest reliability ranging from 0.81 to 0.89 after a retest interval of 1 week [13].

### Statistics

The normality of the input variables was tested by Normal probability plots and the Levene’s test. As most of the variables in this study showed a skewed distribution, the Wilcoxon’s signed rank test was used for the comparison of the variables in the dominant vs non-dominant hand. *P*-values < 0.05 were regarded as statistically significant.

For the comparison of the measured variables between the vibration exposed workers and unexposed referents, the Mann-Whitney U-test was used.

The correlation between the variables in the dominant vs non-dominant hand was checked by the calculation of Spearman rank order correlation coefficients.

The limited number of female workers did not make it possible to statistically compare the results between male and female vibration exposed workers.

All calculations were performed with the Statistical Package for the Social Sciences (IBM SPSS, v. 25.0).

## Results

The temperature perception thresholds for cold and warmth in digits 2 and 5 bilaterally among vibration exposed workers and referents are presented in Table 1.

**Table 1:** Median temperature perception thresholds (TPT, ^o^C) and ranges in the dominant (DH) and non-dominant hand (NDH) in workers and referents, respectively

TPT, ^o^C	All workers (*N* = 47)	*p*-values	Referents (*N* = 18)	*p*-values
Dig 2c DH	25.5 (10–30)	0.08	27.7 (26–31)	0.46
NDH	26.2 (10–31)		28.7 (16–31)	
Dig 2w DH	42.9 (34–50)	0.22	36.7 (34–48)	0.91
NDH	41.6 (34–50)		36.9 (34–47)	
Dig 5c DH	20.3 (10–30)	0.61	25.1 (16–30)	0.91
NDH	22.8 (10–31)		26.5 (10–31)	
Dig 5w DH	43.0 (34–50)	0.25	37.1 (34–48)	0.65
NDH	44.0 (34–50)		37.6 (34–48)	

C = cold thresholds and w = warm thresholds. *P*-values specify the comparison of the studied variables between the DH and NDH in workers and referents

The temperature perception thresholds did not differ significantly between dig 2 and 5 in the dominant vs non-dominant hand in neither workers nor referents. The difference in cold thresholds in dig 2 among the workers was closest to significance (*p* = 0.08) with the better performance in the non-dominant hand. All cold and warmth temperature perception thresholds in dig 2 and 5 in both hands were significantly raised among the workers compared to the reference group (*p* ≤ 0.02).

In Table 2, the outcome of the VPT-tests are shown, after recalculating them to a sensibility index. A sensibility index below 0.8 is considered as pathological.

**Table 2:** Median values and ranges of the sensibility index (SI-index) in dig 2 and 5, respectively, in the DH and NDH in workers and referents.

VPT SI-index	All workers (*N* = 47)	*p*-values	Referents (*N* = 18)	*p*-values
Dig 2 DH	0.83 (0.26–1.23)	0.21	0.94 (0.55–1.24)	0.001
NDH	0.80 (0.18–1.13)		1.08 (0.71–1.49)	
Dig 5 DH	0.79 (0.05–1.14)	0.61	0.94 (0.47–1.42)	0.06
NDH	0.79 (0.15–1.07)		0.99 (0.64–1.54)	

*P*-values specify the comparison of the studied variables between the DH and NDH in workers and referents

No significant difference between the SI-indices in dig 2 and 5, respectively, in the dominant vs non-dominant hand was observed among the exposed workers. A significant difference, however, was noted among the referents who showed a better performance in the non-dominant hand. As evident from the table, the referents showed a significantly higher SI-index in the dominant and non-dominant hand, compared to the exposed workers (*p* ≤ 0.034).

In Table 3, the results for the Purdue Pegboard test reflecting the eye-hand coordination and the muscle strength tests are presented. A significant difference was observed between the dominant vs non-dominant hand for the Purdue Pegboard test in both workers and referents, with a better performance in the dominant hand. The referents performed significantly better (*p* < 0.001) than the workers on the Purdue Pegboard test in both the dominant and non-dominant hand. The handgrip strength and the finger muscle strength tests, however, did not differ significantly between the dominant and non-dominant hand in neither workers nor referents. For the latter tests, no significant differences were observed between workers and referents.

**Table 3:** Median values and ranges for the Purdue Pegboard test, Baseline handgrip (kg), Pinch grip (kg) and 3-Chuck grip (kg) in workers and referents.

Variable	All workers (*N* = 47)	*p*-values	Referents (*N* = 18)	*p*-values
Purdue DH	13.0 (3–16)	0.001	15.5 (13–19)	0.033
Pegboard NDH	12.0 (2–17)		15.0 (9–18)	
Baseline DH	40.0 (8–81)	0.21	42.0 (30–61)	0.11
hand grip NDH	43.8 (6–73)		39.3 (25–68)	
Pinch DH	9.9 (3–15)	0.59	9.2 (6–29)	0.09
grip NDH	9.8 (3–15)		8.5 (6–13)	
3-Chuck DH	8.6 (3–13)	0.67	8.0 (7–13)	0.11
grip NDH	8.4 (2–14)		7.9 (6–14)	

*P*-values specify the comparison of the studied variables between the DH and NDH in workers and referents

In Table 4, the Spearman rank order correlation coefficients between the variables in the dominant and non-dominant hand are presented. The strongest correlation coefficients between the measurements in the dominant vs non-dominant hand, r_s_ around 0.8 or higher, were noted for vibrations perception thresholds in digits 2 and 5 and for the Baseline handgrip, Pinch grip and 3-Chuck grip, indicating a close relationship. Slightly weaker and more varying correlation coefficients were noted for the temperature perception thresholds and for the Purdue Pegboard test.

**Table 4:** Spearman rank order correlation coefficients between the studied variables in the DH vs NDH in workers and referents

Variables	All workers and referents
TPT dig 2c	0.59 p < 0.001
TPT dig 2w	0.60 p < 0.001
TPT dig 5c	0.48 p < 0.001
TPT dig 5w	0.64 p < 0.001
VPT dig 2	0.83 p < 0.001
VPT dig 5	0.88 p < 0.001
Purdue Pegboard	0.71 p < 0.001
Baseline handgrip	0.94 p < 0.001
Pinch grip	0.81 p < 0.001
3-Chuck grip	0.87 p < 0.001

## Discussion

This study is focused on a group of long-term vibration exposed workers (mean exposure time about 16 y), who have been referred to the department of Occupational and Environmental medicine, Sahlgrenska University hospital in Gothenburg because of a suspected vibration injury. As some of the lighter vibrating tools, e.g. screw drivers, were handled solely with the dominant hand, in this case mostly the right hand, we expected the vibration exposure of the dominant hand to be higher than in the non-dominant hand. Accordingly, the higher exposure might give more severe neurosensory signs in the dominant hand, which formed the basis for this study.

The referents showed better warmth (*p* ≤ 0.008) and cold thresholds (*p* ≤ 0.020) in digits 2 and 5 respectively, compared to the vibration exposed workers. Internal comparisons, however, between the dominant and non-dominant hand in all workers and referents, respectively, showed no significant differences (Table 1).

The vibration perception thresholds expressed as Sensibility Index (SI) differed significantly between the dominant and non-dominant hand among the referents, but not among the workers (Table 2). The referents had significantly higher SI-indices in digits 2 and 5 bilaterally, than the vibration-exposed workers (*p* ≤ 0.034), with the better performance in the non-dominant hand.

For e.g. the determination of vibration perception thresholds and temperature perception thresholds the test results might be influenced by differences in reaction time when comparing the dominant and non-dominant hand from the moment when the vibration was perceived until the subject pressed the remote control button [14]. In this study, the rate of change of the vibration amplitude was 3 dB/s during the VPT determination. The temperature decreased or increased by 1 °C per second during the TPT measurements.

Assuming a reaction time of 0.2–0.3 s in normal subjects a possible difference of 0.1–0.2 s in response time between the dominant and non-dominant hand would only have a minor impact on the test results.

Similar findings have been reported by Lindsell and Griffin [8], who did not find any differences in temperature and vibration perception thresholds when comparing the right and left hand in healthy males in working age. An increase in age was usually followed by an increase in thermal thresholds, but the findings were not sufficiently pronounced to motivate an age correction. In a study of 530 healthy volunteers aged 3–79 y, Hilz et al. [15] found no differences in vibration perception thresholds when comparing the right and left body side. Opposite findings have been reported by Ekenvall et al. [16] who observed significantly higher temperature thresholds in the right compared to the left hand in a study of 37 vibration exposed patients with neurological symptoms in the hands.

Nerve conduction measurements of 155 male office and manual workers at an engineering plant showed a longer distal latency in the motor conduction of the median and ulnar nerves over the carpal tunnel in the right hand and similarly also a slightly increased latency in the sensory conduction over the same segment. One explanation can be a higher vibration exposure of the right hand and a higher ergonomic load on this side [17] .

Vibration exposure can activate the autonomic nerve system and affect the blood flow in both hands, even if only one hand is vibration exposed. Such blood flow changes have probably only a minor impact on the distal neuropathy, which was diagnosed in hands and fingers of the exposed workers. The main reason for the distal neuropathy is probably a direct effect of the vibration transmission to the nerves and mechanoreceptors (e.g. Meissners’s and Pacinian corpuscles) in the fingers. Reported histological findings include e.g. demyelinating neuropathy, loss of nerve fibers, fibrosis of the perineurium as well as dysfunction of mechanoreceptors in the skin of the fingers [18].

In this study, there were no workers who only worked with light vibrating tools held in the dominant hand. The dominant vibration exposure from heavier vibrating tools, which are held in both hands, has probably had a significantly greater importance for the development of neurosensory symptoms and signs, than the exposure from lighter tools. Thus, the additional vibration exposure of the dominant hand from smaller and lighter vibrating tools was probably not large enough to give a significant difference in neurosensory symptoms and signs when comparing the dominant and non-dominant hand.

The Purdue Pegboard test showed a better performance in the dominant hand compared to the non-dominant hand in both workers and referents. The better performance in the dominant hand is probably due to a better-developed eye-hand coordination in the dominant hand. As evident from Table 3, the referents performed significantly better (*p* < 0.001) in both the dominant and non-dominant hand compared to the vibration exposed workers.

In our study, no differences in hand and finger grip strength were observed between the dominant and non-dominant hand in neither workers nor referents. Several investigators, however, have reported a difference in hand grip strength in the dominant and non-dominant hand in non-vibration exposed subjects. In a Turkish study, the dominant hand of right-handed subjects was significantly stronger than the non-dominant hand, but for left-handed subjects no significant difference in grip strength was detected [19]. A number of other studies have shown similar results. In a review of 10 studies examining the difference in grip strength in dominant and non-dominant hands, right-handed subjects were stronger on the dominant side [20]. For left-handed subjects the results were equivocal. In a study of 310 males and females, Petersen et al. [21] found that right-handed subjects were about 13% stronger in the right hand than in the left hand while the results were equivalent for left-handed subjects. The findings are in favor of the hypothesis that the dominant hand is about 10% stronger than the left hand [21]. Small but significant differences up to 3% have been observed between hand grip strength in dominant and non-dominant hands in right handed participants for tests of maximum voluntary contraction of the first interosseous muscle, power grip strength and pulp-to-pulp pinch strength in a study of 83 healthy subjects [22]. However, no significant differences were noted between the dominant and non-dominant hand in left-handed subjects for all tests. In our study, we had expected to find stronger hand and finger muscle strength test results among the vibration exposed workers. There are, however, two factors that may have influenced this comparison. The referents were about 10 years younger than the workers, which may have narrowed the strength gap. Furthermore, the long-term vibration exposure (mean 16 y) may have weakened the muscle strength in the hands and fingers of the workers and thereby equalized the assumed difference in strength.

Sensitivity to light touch and pressure can be checked with the Semmes Weinstein’s monofilament test, testing the function of the Aß-nerves, which also transmits the signals from vibration exposure. In a study of 50 volunteers, tests on the entire palm showed no difference in more than half of the subjects when comparing the left and right hand. The non-dominant side showed superior sensibility in approximately one third of the subjects. In less than one sixth of the subjects, superior sensibility was noted in the dominant hand [23]. Similar results have been reported by Hage et al. [24] testing the sensibility of both index fingers with Semmes Weinstein’s monofilament test in a study of 130 active subjects aged 7 to 76 years. No difference between the left and right side was found in 76 subjects. The non-dominant side had a superior sensibility in 35 subjects and the dominant side a superior sensibility in the remaining 19 cases.

Strong correlation coefficients between the test values in the dominant vs non-dominant hand hand were observed for several of the tests in our study, Baseline handgrip strength, Pinch grip, 3-Chuck grip and VPTs in dig 2 and 5, respectively in both groups (Table 4). Somewhat lower correlation coefficients were noted for temperature perception thresholds and the Purdue Pegboard test,

## Conclusions

Several studies have reported differences as regards neurophysiological test results in the dominant vs non-dominant hand in long-term vibration exposed workers. In this study, similar minor differences between the dominant and non-dominant hand were noted for the Purdue Pegboard test in both workers and referents. Despite a probably higher vibration exposure in the dominant hand (mostly the right hand), however, quite similar test results were noted for VPTs, TPTs, Baseline handgrip, Pinch grip and 3-Chuck grip when comparing the dominant and non-dominant hand in the exposed workers. In case of lack of time and financial obstacles, neurological tests in solely the dominant hand, will probably satisfactory reflect the conditions in the non-dominant hand.

## Data Availability

The dataset supporting the conclusions of this article is included within this manuscript.

## Abbreviations

DH: Dominant hand
NDH: Non-dominant hand
SI: Sensibility Index
TPT: Temperature perception thresholds
VPT: Vibration perception thresholds
VWF: Vibration white fingers
