# Correction to: Vibration induced injuries in hands in long-term vibration exposed workers

**DOI:** 10.1186/s12995-019-0245-x

**Published:** 2019-08-23

**Authors:** Lars Gerhardsson, Mats Hagberg

**Affiliations:** Occupational and Environmental Medicine, University of Gothenburg, Medicinaregatan 16, Box 414, SE-405 30 Gothenburg, Sweden

**Correction to: J Occup Med Toxicol**

**https://doi.org/10.1186/s12995-019-0242-0**

Following publication of the original article [1], it has been brought to our attention that an error was slipped into the article’s title.

- Initially published title:

Style: J of occupational medicine and toxicology vibration induced injuries in hands in long-term vibration exposed workers

- Corrected title:

Vibration induced injuries in hands in long-term vibration exposed workers

The original article has been corrected.

## References

[1] Gerhardsson L, Hagberg M (2019). Vibration induced injuries in hands in long-term vibration exposed workers. J Occup Med Toxicol.

